# Endoscopic Endoluminal Radiofrequency Ablation and Single-Operator Peroral Cholangioscopy System (SpyGlass) in the Diagnosis and Treatment of Intraductal Papillary Neoplasm of the Bile Duct: A Case Report and Literature Review

**DOI:** 10.3389/fmed.2021.675720

**Published:** 2021-09-16

**Authors:** Wei Tang, Jian-Guo Qiu, Xu-Fu Wei, Heng Xiao, Xin Deng, Shao-Dong Wang, Cheng-You Du, Qiao Wu

**Affiliations:** ^1^Department of Hepatobiliary Surgery, The First Affiliated Hospital of Chongqing Medical University, Chongqing, China; ^2^Department of Urology, Xiangya Hospital, Central South University, Changsha, China

**Keywords:** intraductal papillary neoplasm of the bile duct, endoscopic endoluminal radiofrequency ablation, single-operator peroral cholangioscopy system, spyglass, case report

## Abstract

**Background:** Intraductal papillary neoplasm of the bile duct (IPNB) is a rare biliary benign tumor with atypical clinical features and is frequently misdiagnosed. Its treatment is limited and surgical resection is thought to be the only therapeutic option in patients with IPNB. With the aim of increasing the early diagnosis rate of IPNB and providing more therapeutic options for surgeons, we innovatively put forward the concept of combined utilization of SpyGlass and endoscopic endoluminal radiofrequency ablation (ERFA) in the diagnosis and treatment of IPNB.

**Case Presentation:** An 85-year-old woman was referred to our hospital due to right upper quadrant abdominal pain. The image examinations indicated suspicious filling defects at the upper common bile duct. Further evaluation of SpyGlass cholangioscopy showed multiple reddish villous lesions at the left hepatic duct, and SpyBite biopsy under direct visualization demonstrated papillary low-grade dysplasia. In consideration of the advanced age and preference of the patient, the novel ERFA therapy was performed. The procedure was successful without periprocedural complications; the patient recovered uneventfully and was discharged 2 days after the operation. Upon follow-up, the patient was asymptomatic and in good physical condition at 8 months postoperatively.

**Conclusion:** Preliminarily, we demonstrate that the strategy of a combination of SpyGlass and ERAF seems to be a promising, feasible, well-tolerated, and safe management for patients with IPNB. However, more data with larger patient volumes are needed to evaluate its outcomes further.

## Background

Intraductal papillary neoplasm of the bile duct (IPNB) is a rare biliary benign tumor characterized by multiple papillary adenomas of intrahepatic or extrahepatic bile ducts with proliferated bile duct epithelial cells (1). Since first presented in 1959 (2), more than 300 IPNB cases have been reported thus far. Its clinical features are atypical, such as abdominal pain, jaundice, recurrent episodes of cholangitis, and the presence of biliary stones or infection. Recurrent biliary tract infection can even lead to secondary biliary cirrhosis and liver failure (3–5). As IPNB is a relatively rare tumor, and its atypical clinical manifestations, initial and differential diagnoses, are usually difficult and complex. Although various diagnostic modalities have been used, IPNB is still often misdiagnosed as biliary stones and remains unrecognized for long. Subsequently, patients with IPNB experience aggravated papillomatosis and have to undergo extensive surgery after diagnosis (6). Currently, the management of IPNB is limited, and surgical resection is thought to be the only therapeutic option for patients with IPNB, such as liver resection and liver transplantation for intrahepatic lesions, and cholecystectomy and pancreaticoduodenectomy for extrahepatic lesions. In this case study, with the aim of increasing the early diagnosis rate and providing more therapeutic options for surgeons, we reported our experience of an IPNB case in which the patient was diagnosed with the help of the single-operator peroral cholangioscopy system (SpyGlass) and was primarily treated by endoscopic endoluminal radiofrequency ablation (ERFA).

## Case Presentation

**Figure 1:**
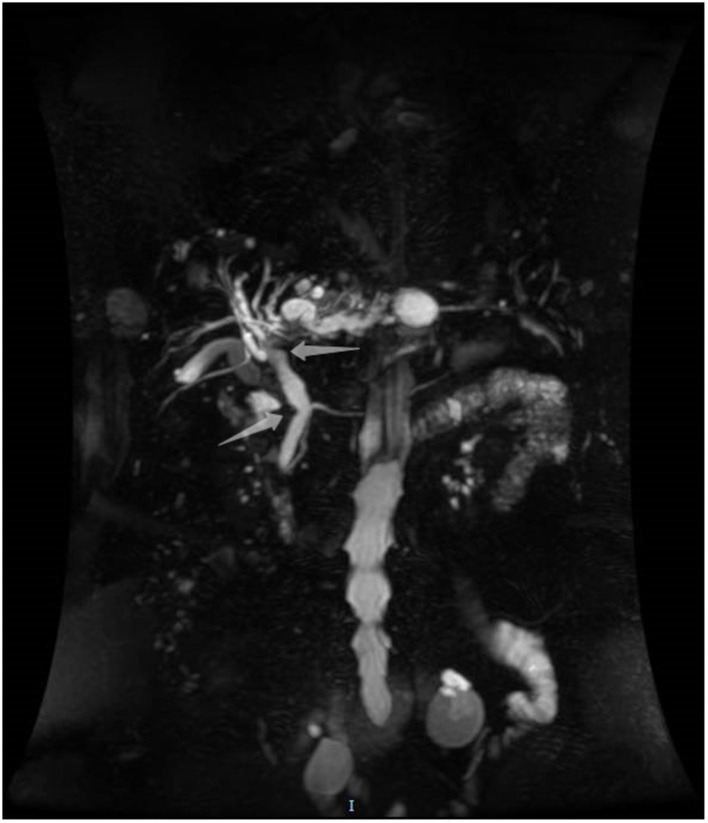
Magnetic resonance cholangiopancreatography showing a suspicious filling defect at the upper common bile duct.

**Figure 2:**
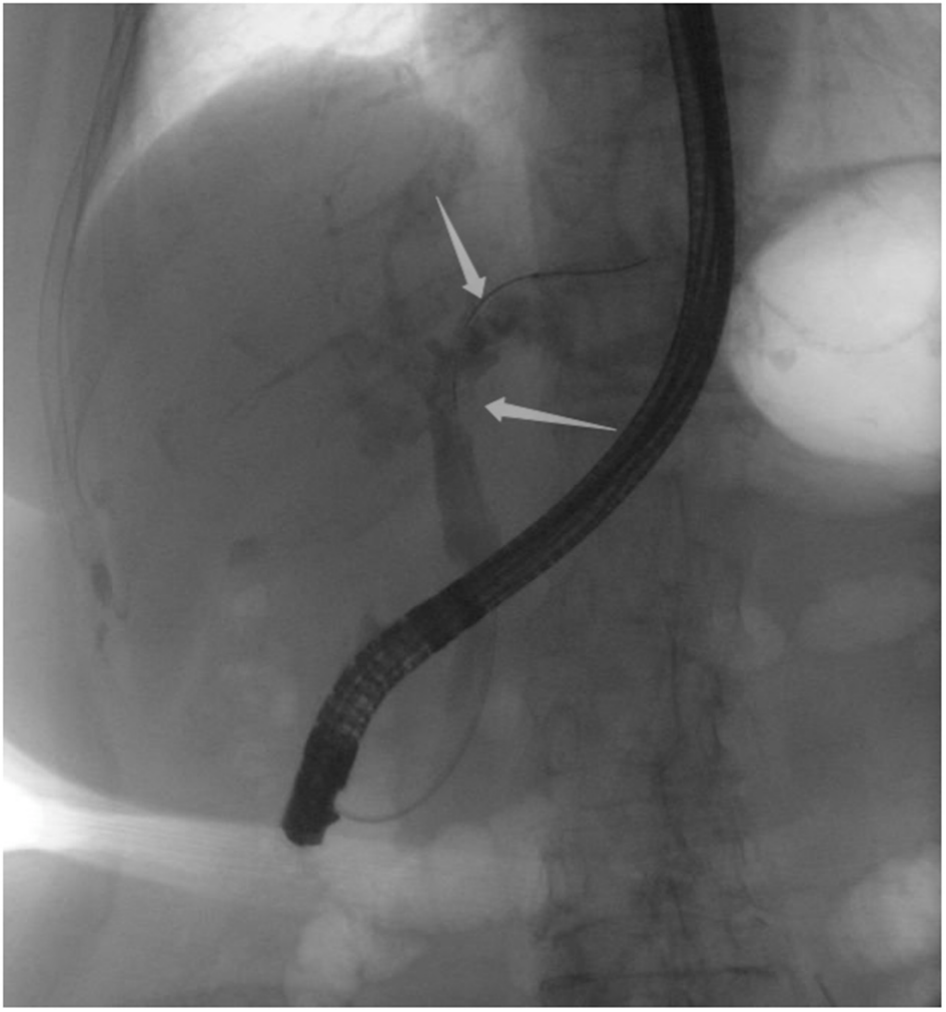
Endoscopic retrograde cholangiopancreatography indicating multiple filling defects at the left hepatic duct and the distal bile ducts were dilated.

**Figure 3:**
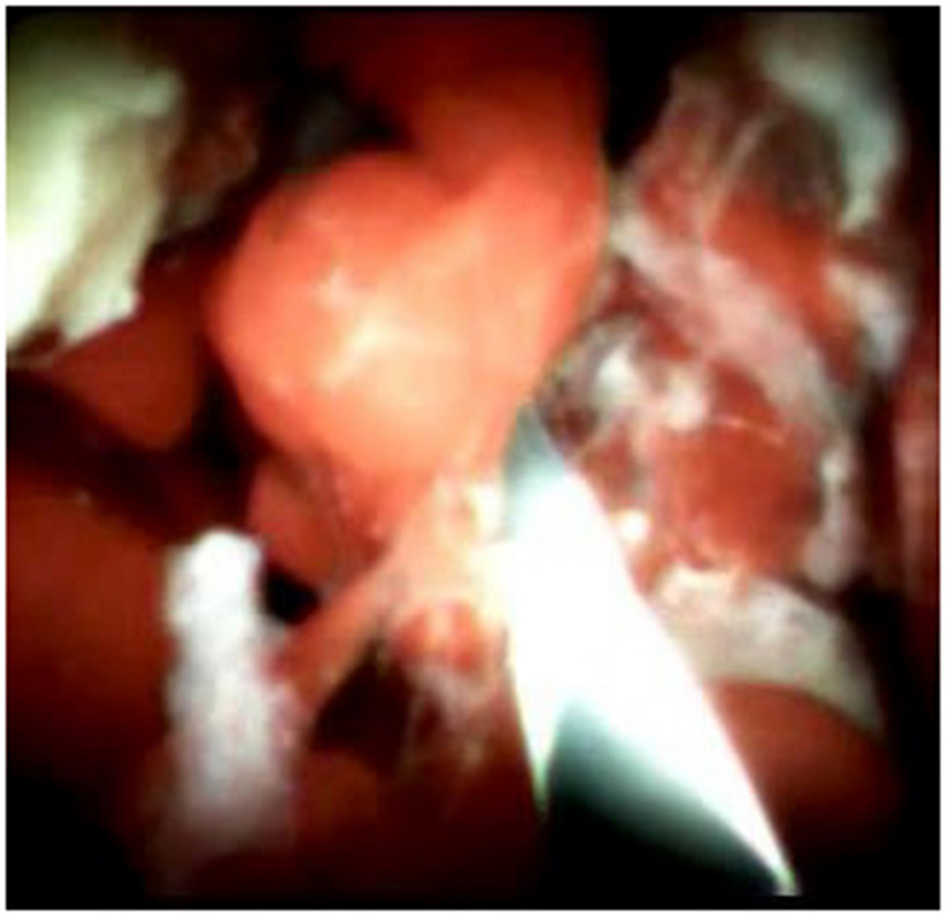
SpyGlass cholangioscopy revealing multiple reddish villous lesions with fine vascular cores on the surface in the left hepatic duct lumen.

**Figure 4:**
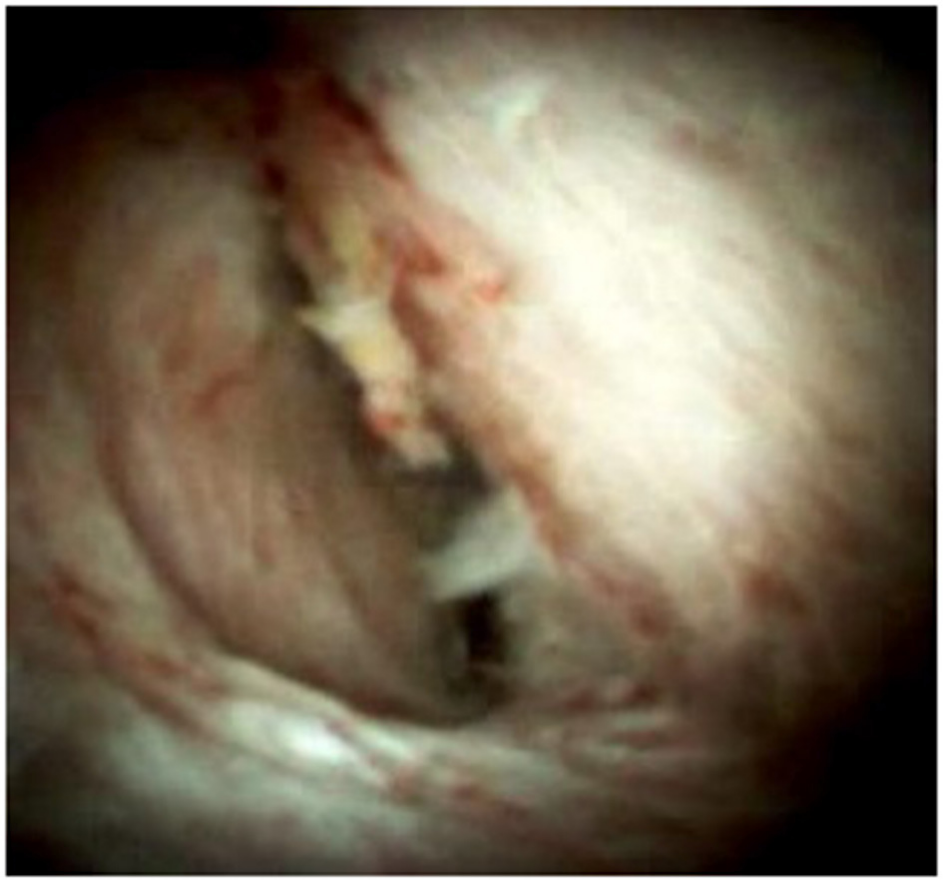
Repeat SpyGlass cholangioscopy confirming no residual villous lesions.

An 85-year-old woman was admitted to our surgical department due to 3-week history of recurrent right upper quadrant abdominal pain, right shoulder radiating pain, and nausea on May 31, 2020. Her medical history included primary hypertension, and she was on lisinopril therapy. Physical examinations revealed mild tenderness at the right upper quadrant of the abdomen without other signs. Hematological examinations indicated that routine blood test results were normal. Liver function tests showed the γ-glutamyltransferase was 258 IU/L. Bilirubin, alanine aminotransferase, asparagine aminotransferase, and albumin levels were normal. Mild hypokalemia was observed in the patient. With regard to the tumor markers, the results indicated a slightly elevated carbohydrate antigen 19–9 level of 35.3 U/ml, carbohydrate antigen 72–4 level of 9.8 U/ml, and carcinoembryonic antigen level of 10.5 ng/ml. Other tumor markers, including α-fetoprotein and cancer antigen 125, were observed to be normal. Tests for hepatitis B and C viruses were negative. Examinations for serum amylase level, stool routine, and urine routine were unremarkable. Abdominal ultrasonography, enhanced CT, and magnetic resonance cholangiopancreatography (MRCP) indicated that the common bile duct was slightly dilated, while the left hepatic duct and the left intrahepatic ducts were expanded with multiple left localized hepatolithiasis. In addition, MRCP revealed a suspicious filling defect at the upper common bile duct (Figure 1). To diagnose definitely, further evaluation was needed. Thus, on June 3, endoscopic retrograde cholangiopancreatography (ERCP) with SpyGlass Direct Visualization System (Boston Scientific, Natick, MA, USA) was performed for the patient. The papilla was normal. Selective deep cannulation of the common bile duct was achieved. The cholangiogram showed that multiple filling defects at the left hepatic duct and the distal bile ducts were dilated (Figure 2). After papillary sphincterotomy and balloon dilatation were performed, the SpyGlass cholangioscopy was inserted into the bile duct for direct visualization of the tissue through a channel of an ERCP scope. Direct visualization revealed multiple reddish villous lesions with fine vascular cores on the surface in the left hepatic duct lumen (Figure 3). No mucin material was observed. The left hepatic duct lumen was obviously narrow with a stone in it. While the rest of the biliary tree was normal. The stone was extracted using a basket, and targeted tissue samples were obtained with the SpyBite biopsy forceps (Boston Scientific) under direct visualization. At the end of the procedure, internal biliary drainage was performed using a plastic stent to decompress the left ductal system. The procedure was performed in 58 min, without periprocedural complications. Histopathology of the biopsy specimen showed papillary low-grade dysplasia. Based on these findings, a preoperative diagnosis of IPNB was established. However, the patient refused a radical resection surgery. In consideration of the advanced age and treatment preference of the patient, ERFA with SpyGlass was offered as a primary treatment after reviewing the literature. On June 10, a second ERCP with SpyGlass was performed. The biliary drainage stent was extracted. After confirming the localization and extension of the biliary lesions using SpyGlass cholangioscopy (Supplementary Video 1), the ERFA catheter (Lide Electronics, Mianyang, Sichuan, China) with a 200 cm working length was then inserted into the papilla of Vater over a guide wire through a duodenoscope working channel, the ablation catheter was connected to a radiofrequency generator (Lide Electronics) to deliver the power over a duration of 15–20 min for each application. The ERFA energy was applied in a diameter manner (radiofrequency ablation diameter: 0.8 cm) along the entire length of the biliary lesions for 2 min totally. After direct visualization of the cholangioscopy confirming complete damage of the lesions (Figure 4 and Supplementary Video 2), the necrotic tissue was extracted using a basket. Lastly, a nasobiliary drainage tube was placed. The duration of the procedure was 106 min. Postoperative treatment included antibiotics, somatostatin, proton pump inhibitor, and hemostatic drug, etc. The patient recovered uneventfully and was discharged 2 days after the operation. Upon follow-up, the patient was asymptomatic and in good physical condition at 8 months postoperatively.

## Discussion

Intraductal papillary neoplasm of the bile duct, based on the latest WHO classification in 2010, is defined as an epithelial tumor characterized by a papillary or villous neoplasm covering delicate fibrovascular stalks occurring in the bile ducts (7). It is histologically similar to the intraductal papillary mucinous neoplasm of the pancreas (IPMN-P), and it has been suggested that IPNB is the biliary counterpart of IPMN-P for similar histopathologic features (8). Based on the epithelial nature and structure, IPNB is classified into four histology subtypes: pancreatobiliary (most common), gastric, intestinal, and oncocytic (least common) (9). According to the degree of cytologic and structural atypia, IPNB is defined as IPNB with low-/intermediate-grade intraepithelial neoplasia, IPNB with high-grade intraepithelial neoplasia, and IPNB associated with invasive carcinoma. In addition, IPNB is also classified as mucin-hypersecreting type and non-mucin-producing type, depending on the presence or absence of mucin hypersecretion (10). Though it is histologically categorized as benign neoplasm, IPNB is still considered to be a premalignancy disease with malignancy potential, due to a high incidence of recurrence after resection and a high malignant transformation rate of about 41% according to previous reports (10, 11). The definitive cause and pathogenesis of IPNB remain unclear, recurrent infection and bile stasis induced by hepatolithiasis or clonorchiasis and pancreatic juice reflux likely contribute to the chronic inflammation and extensive proliferation of biliary epithelial cells, subsequently leading to mucosal changes and IPNB (11, 12).

Owing to the atypical clinical manifestations and non-specific image features of IPNB, an accurate preoperative diagnosis is usually hard to reach. The most common clinical symptoms include recurrent and intermittent abdominal pain, repeated relapsing cholangitis, and jaundice (13). Ultrasound and CT might indicate the presence of soft tissue densities in the intrahepatic or extrahepatic ducts with proximal dilation of the bile ducts. MRCP and ERCP might reveal ductal dilation with or without undefined filling defects. However, no special diagnostic or radiologic feature has been described. As for the tumor markers, although the CA19-9 level could be higher in some patients with IPNB, it does not allow a precise differentiation between benign and malignant tumors. They are not pathognomonic of this condition. Thus, preoperative diagnosis of these biliary lesions is challenging for surgeons. Fortunately, since 2005, the single-operator peroral cholangioscopy system (SpyGlass), which is characterized by stable image, wide field of view, and consistent orientation of the biopsy channel, has been available in biliary diagnostic and therapeutic procedures. SpyGlass allows not only direct visualization of the lesions to pinpoint their location but also tissue biopsy of the lesions to facilitate pathologic diagnosis. As the previous study reported, the sensitivity and specificity of SpyBite biopsies were 76.5 and 100.0%, respectively, whereas the sensitivity of conventional ERCP brushing biopsies was 5.8% (14). The early diagnosis rate of the biliary tumor was highly increased. In general, the advantages of SpyGlass were thought to be the direct visual impression of biliary lesions, which could help diagnose the exact localization of the lesions and determination of the tumor extension for subsequent surgical planning in order to perform a radical resection and the high sensitivity of SpyBite biopsies under direct visualization. Moreover, for patients with IPNB, the main points of endoscopic diagnosis include the classic fish-eye appearance of the ampulla of Vater, the presence of thick mucin material in the bile ducts, and the existence of reddish papillary protrusions involving the bile ducts. With regard to the indications of the application of SpyGlass, our experience suggested that SpyGlass should be considered as a routine examination to improve the early diagnosis rate of biliary tract disease, such as IPNB in the following two situations: (1) patients with indeterminate biliary lesions or filling defect and (2) patients with unexplained recurrent episodes of cholangitis or biliary stones.

Regarding the treatment of IPNB, radical resection with negative margins, such as bile duct resection with or without major hepatectomy, pancreaticoduodenectomy, and liver transplantation, is thought to be the curative therapy, while palliative treatment includes transhepatic percutaneous drainage, endoscopic nasobiliary drainage, and chemotherapy. Radiofrequency energy, which induces coagulative necrosis of tumor tissue by a controlled temperature rise, has been widely employed as a method of tumor ablation. As reported by the previous studies (15–17), radiofrequency ablation has been adopted in a few IPNB cases and achieved promising results. In our case, considering that the successful results of radiofrequency ablation in malignant biliary obstruction, and previous patients with IPNB, as well as, a limited alternative to traditional surgical treatment, we put forward ERFA as a primary treatment modality for the patient with IPNB. Combined with the advantages of localization function and real-time treatment effectiveness evaluation of SpyGlass, ERFA appears to be a promising therapy in patients with IPNB. In our opinion, the following were thought to be the advantages of ERAF combined with SpyGlass. First, as a minimally invasive procedure, it provides advantages of less pain, fast recovery, and fewer side effects. Second, in the circumstance of the scarcity of treatment therapy, it offers more options for patients with IPNB, especially the patients with advanced age or bad general status. Third, the integration mode of diagnosis and treatment for patients with IPNB.

## Conclusion

To our knowledge, this is the first study to establish the concept of combined SpyGlass and ERAF as a potential alternative to surgical treatment in patients with IPNB. With the present work, we preliminarily demonstrated that the strategy of the combination of SpyGlass and ERAF seemed to be a promising, feasible, well-tolerated, and safe management for patients with IPNB. Furthermore, whether this strategy is universally applicable for other benign biliary tumors is interesting and is worth investigating deeply. However, more data with larger patient volumes are needed to evaluate the short-term and long-term outcomes further.

## Data Availability Statement

The raw data supporting the conclusions of this article will be made available by the authors, without undue reservation.

## Author Contributions

WT and J-GQ reviewed the literature and contributed to manuscript drafting. X-FW and HX analyzed and interpreted the imaging findings and reviewed the literature. XD and S-DW collected the data and made the figures. C-YD and QW designed the study, offered suggestions for this study, and were responsible for the revision of the manuscript for important intellectual content.

## Funding

This study was supported by the Basic Research and Frontier Exploration Project of Chongqing Science and Technology Commission (cstc2017jcyjBX0010), the Basic Research and Frontier Exploration Project of Chongqing Science and Technology Commission (cstc2018jscx-msybX0133), and the National Natural Science Foundation of China (81702408).

## Conflict of Interest

The authors declare that the research was conducted in the absence of any commercial or financial relationships that could be construed as a potential conflict of interest.

## Publisher's Note

All claims expressed in this article are solely those of the authors and do not necessarily represent those of their affiliated organizations, or those of the publisher, the editors and the reviewers. Any product that may be evaluated in this article, or claim that may be made by its manufacturer, is not guaranteed or endorsed by the publisher.

## Supplementary Material

Supplementary Video 1 SpyGlass cholangioscopy showing multiple reddish villous lesions with fine vascular cores on the surface in the left hepatic duct lumen.

Supplementary Video 2 SpyGlass cholangioscopy showing no residual villous lesions after endoscopic endoluminal radiofrequency ablation.

## Abbreviations

ERCP: endoscopic retrograde cholangiopancreatography
ERFA: endoscopic endoluminal radiofrequency ablation
IPNB: intraductal papillary neoplasm of the bile duct
MRCP: magnetic resonance cholangiopancreatography.
